# Proximal Tibia Bone Graft: An alternative Donor Source especially for Foot and Ankle Procedures

**DOI:** 10.5704/MOJ.1503.016

**Published:** 2015-03

**Authors:** TY Jia, S Gurmeet, A Asni, R Ramanathan

**Affiliations:** Department of Orthopedics, Hospital Raja Permaisuri Bainun, Perak, Malaysia

**Keywords:** Bone graft, Proximal tibia bone graft, Foot and ankle procedures

## Abstract

Bone graft is essential in various orthopaedic procedures. Among the many donor sites for harvesting autologous bone graft, the iliac crest has been the most commonly used. However, for foot and ankle procedures the proximal tibia has gained popularity as an alternative donor site due to its anatomic proximity to the primary surgical site. In this article we evaluated the possible complications associated with harvesting proximal tibia bone graft. Our study showed the low incidence of morbidity in harvesting proximal tibia bone graft, thereby providing a good alternative donor for foot and ankle procedures.

## Introduction

Bone graft is commonly used in orthopaedic surgery to fill up a bony defect. It can be autograft, allograft or synthetic bone graft. An ideal bone-graft substitute must possess various properties, including osteoconductive, osteoinductive, progenitor cells for osteogenesis and structural integrity. Additionally, the bone graft must be able to integrate with the host tissue.

The presence of multipotent mesenchymal stem cells in the periosteum places autograft superior to both allograft and synthetic bone graft in repair of large structural bone defect4. Among the common donor sites for harvesting autologous bone graft, the iliac crest has been the most commonly used for orthopaedic procedures. However, the proximal tibia has gained popularity as an alternative donor site due to its anatomic proximity to the foot and ankle, as stated in the study by Jochen Hahne *et al*^5^. Besides that, other possible advantages of proximal tibia bone grafts are the availability of sufficient volume of bone graft and low donor-site morbidity. Several studies have reported a complication rate of 1-4% from harvesting proximal tibia bone graft, which is less than the rate for iliac bone graft harvesting^1,2,3^. The most frequent donor site complications associated with iliac crest bone graft are nerve injury and hematoma^7,8^. In the present study, the incidence of proximal tibia bone graft donor-site related morbidity was assessed.

## Materials and Methods

We retrospectively reviewed data obtained from the medical records or telephone interview, of nine patients who had proximal tibia donor site for bone grafting as an adjunct to orthopaedic surgical procedures at Hospital Raja Permaisuri Bainun, Ipoh during the period from 1st of January 2014 to 1st of September 2014. All data were collected irrespective of the age, gender, race and smoking habits of the subjects in the study. The exclusion criteria included patients receiving autologous bone graft or synthetic bone graft. This study's aim was to assess the possibledonor site morbidities including pain (acute and chronic), parasthesia, infection rate, incidence of fracture, incidence of hematoma and abnormal scar formation, as well as other abnormal swelling at the donor site.

Among the nine documents analysed, six patients were male and 3 female. The mean age of the patients was 51 years. The commonest orthopaedic procedure was ankle fusion (7 cases), and one each of subtalar fusion and distal medial tibial locking plate for non-union of a fracture in the distal third of left tibia.

### Procedure

Proximal tibia bone graft can be harvested using lateral or medial approach. Herford *et al* studied the amount of cancellous bone and related anatomy via the lateral and medial approaches and concluded that an equal amount of bone graft was available from either approach, but that the medial approach offered an easier technique and possibly safer dissection^6^.

**Fig. 1:**
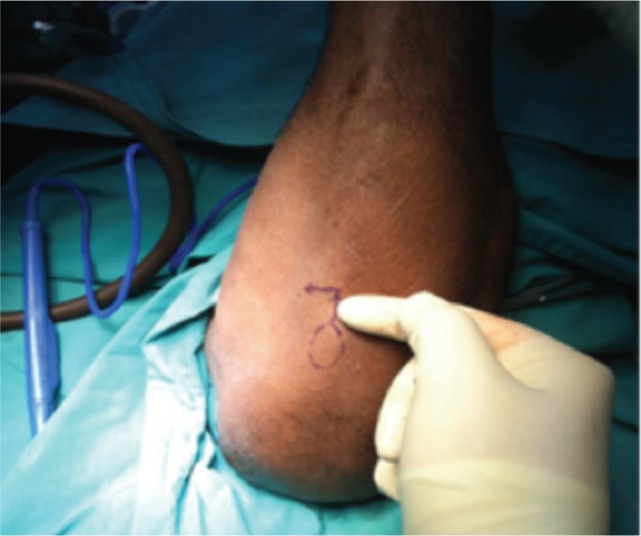
Landmark of skin incision for proximal tibia bone graft.

**Fig. 2:**
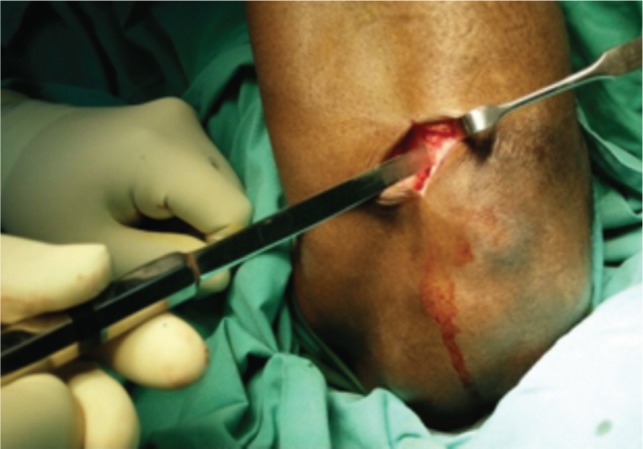
Osteotome size 1cm x 1cm is used to osteotomise the proximal tibia.

**Fig. 3:**
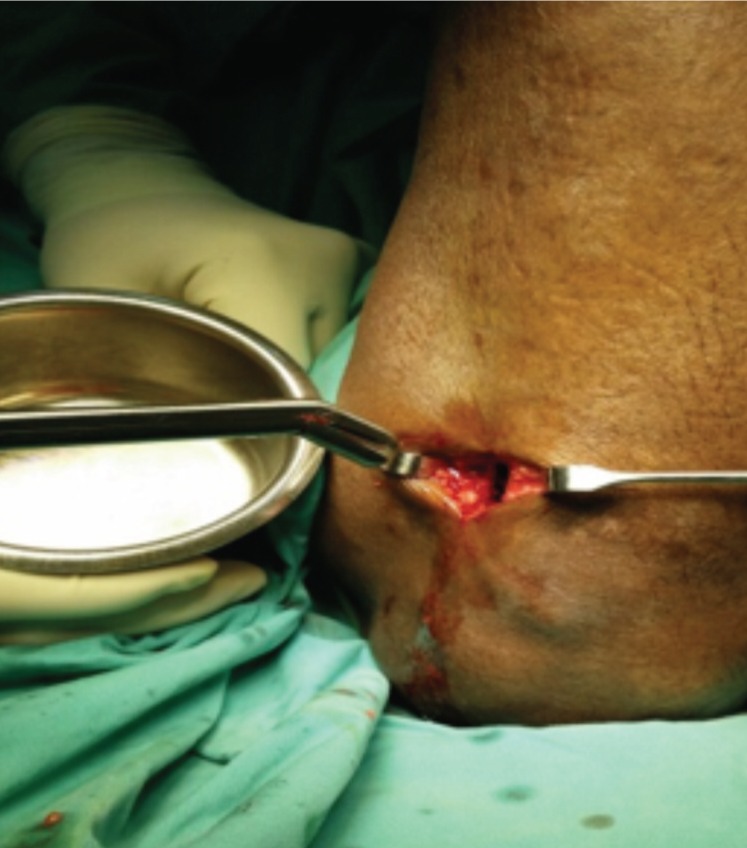
Bone graft is harvested using right angled pituitary forceps.

In this study, proximal tibial bone graft had been harvested in all cases using the medial approach under tourniquet control. A vertical skin incision 1-1.5cm was made on the anterior surface below the knee joint on the medial aspect, skin was retracted and the periosteum stripped using periosteum elevator. The landmark was 2 cm below and medial to the tibia tuberosity, on the flat surface of the tibia bone [Fig 1]. Cortical bone was osteotomized using osteotome size 1cm x 1cm [Fig 2]. Bone graft was then carefully harvested by using curette and angled pituitary forceps [Fig 3]. The amount of bone graft was harvested as required for the primary surgical procedure [Fig 7].

**Fig. 4:**
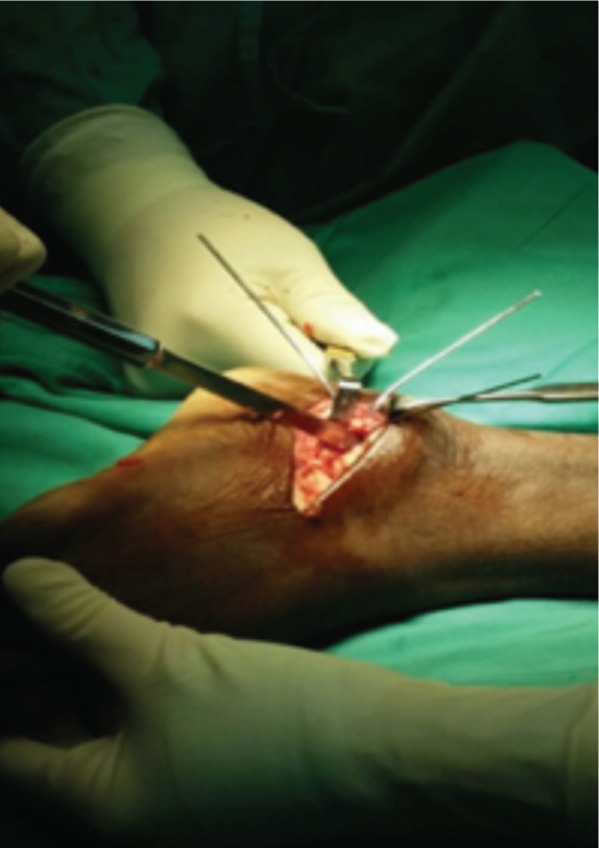
Subtalar joint is explored and cleaned and curretted.

**Fig. 5:**
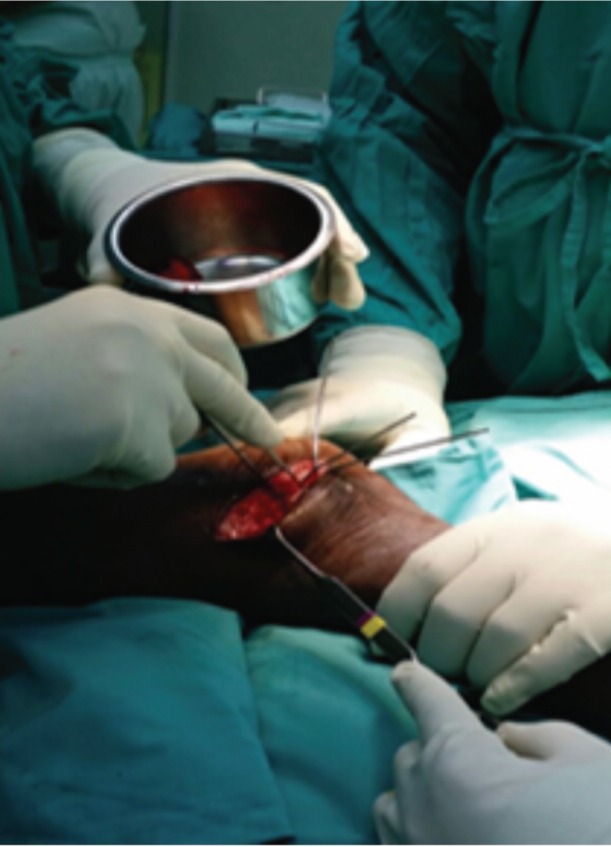
Harvested bone graft is placed in the subtalar joint.

**Fig. 6:**
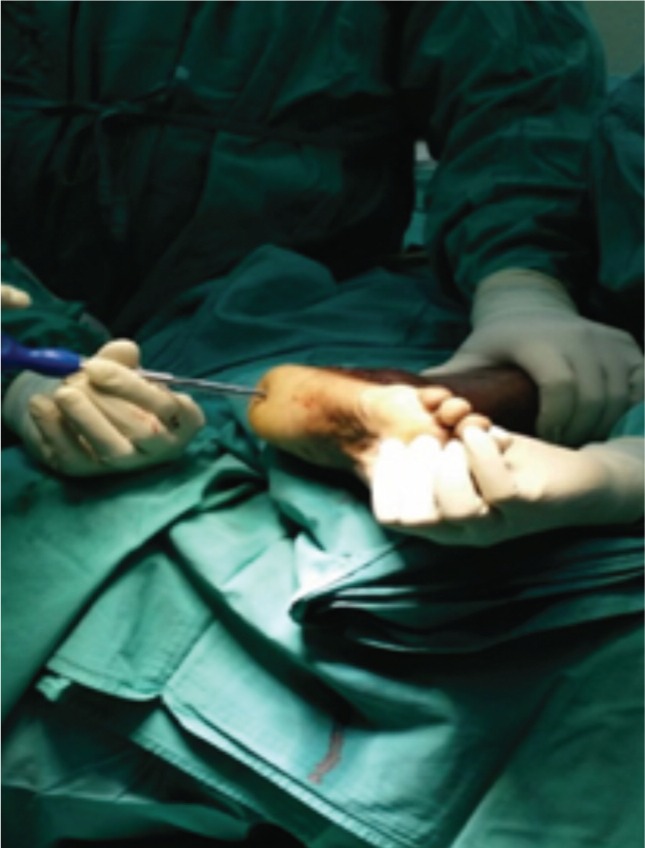
Subtalar fusion done with cannulated screw insertion.

**Fig. 7:**
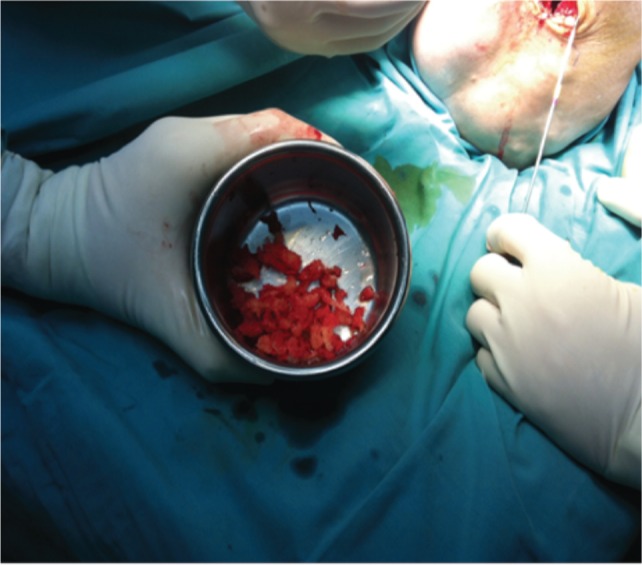
Considerable amount of cancellous bone can be harvested from proximal tibia bone.

Post-operatively, all patients were advised strict non-weight bearing ambulation in view of their primary surgery which was on the ipsilateral limb. Post-operatively, all patients were advised strict non-weight bearing ambulation in view of their primary surgery which was on the ipsilateral limb. They were then followed-up in outpatient clinic in 4 to 6 weeks.

## Results and Discussion

In foot and ankle surgery, the most frequent indication for the use of bone graft including arthrodesis and treatment of fracture non-union^5^. Despite the increasing availability of allograft bone and synthetic bone graft, autogenous bone graft is used frequently as fresh autogenous bone has osteoinductive, osteoconductive and osteogenic properties. Futhermore, infectious and immunologic complications are avoided. Despite iliac crest being the most commonly used site for harvesting bone graft, proximal tibia is an alternativesite for harvesting bone graft preferredby the foot and ankle team in Hospital Ipoh. The main advantage of harvesting bone graft from this site is due to its anatomical proximity to the primary operation site. It is also more convenient in terms of preparing the patient pre-operatively as well as intra-operatively. Besides that, the fact that tibia bone has a subcutaneous surface, which renders it easily accessible regardless of the build of the patient, and the graft is obtained without having to alter the position of the patient during surgery.

The nine patients in this had undergone the primary surgery with additional bone graft harvested from proximal tibia between 1st of January 2014 to 1st of September 2014.. The complications assessed included acute and chronic postoperative pain, parasthesiae at the bone graft donor site, incidence of hematoma formation, incidence of fracture, infection rate and scar formation, and any other abnormal swellings at the donor site at the proximal tibia.

Based on the results recorded in the documents, no incidence of hematoma formation, parasthesia, infection, abnormal swelling or fracture had been reported at bone graft donor site. This is consistent with findings in a cadaveric study by P. Vittayakittipong et al. which showed that the strength of decancellated tibias and intact tibias after harvesting cancellous bone graft were not different^1,3^.

However, acute post-operative pain was recorded in all the patients, the pain intensity assessed by using VAS score. All nine patients had tolerated the pain well with pain score of 1-2/10. Upon discharge, all patients had pain score of less than 3/10. A survey was carried out to inquire about the pain score at bone graft donor-site at the first follow-up outpatient visit, all patients reported pain score of 0/10.

Of the nine patients, five were allowed to fully weight bear on the third month, two patients at fourth month and two remaining patients were at the time of this report still nonweight bearing due to absence of clinical and radiological evidence of union. Overall, proximal tibia bone graft showed good union rate. It is supported by several other studies which reported successful clinical results and similar efficacy of iliac crest and tibia bone grafts in promoting fusion in foot and ankle procedures with low rate of nonunions^5^.

## Conclusion

This is a small sample retrospective study which has shown that there was low incidence of donor-site related morbidity from proximal tibia bone grafting, except for mild pain which was tolerable and relieved by simple analgesia. Proximal tibia, therefore, offers a good alternative site for harvesting bone graft especially for ipsilateral foot and ankle surgery.
